# Supplementary material for: Effect of Flowering Time-Related Genes on Biomass, Harvest Index, and Grain Yield in CIMMYT Elite Spring Bread Wheat
Source: Biology (Basel). 2021 Sep 1;10(9):855. doi: 10.3390/biology10090855 (PMC8471161; doi:10.3390/biology10090855)
Supplement: Supplementary file 1 [file biology-10-00855-s001.zip › Suppl. Figure S1.pptx]

## Slide 1
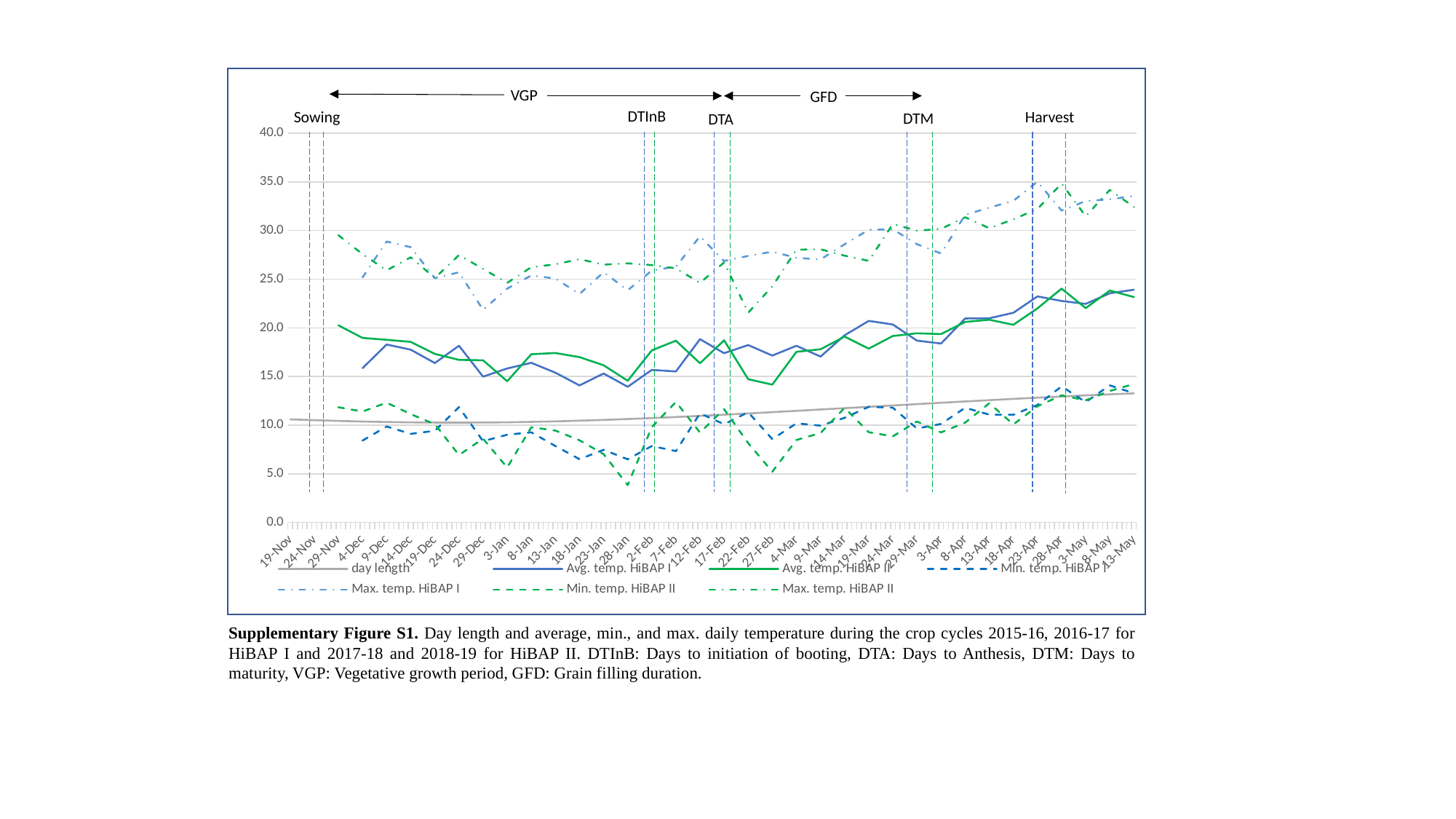

VGP
GFD
DTInB
Harvest
Sowing
DTM
DTA
### Chart
| Category | | | | | | | |
|---|---|---|---|---|---|---|---|
| 44519 | 10.594583651438937 | None | None | None | None | None | None |
| 44524 | 10.50798181700659 | None | None | None | None | None | None |
| 44529 | 10.43255333337022 | None | 20.256999999999998 | None | None | 11.833 | 29.506 |
| 44534 | 10.369439270325298 | 15.853750000000002 | 18.964000000000002 | 8.412500000000001 | 25.175 | 11.396 | 27.57 |
| 44539 | 10.319664572000502 | 18.287 | 18.768 | 9.86 | 28.869999999999997 | 12.306000000000001 | 25.904999999999994 |
| 44544 | 10.284087909447077 | 17.752 | 18.557499999999997 | 9.1 | 28.28 | 11.126 | 27.237000000000002 |
| 44549 | 10.263355814156819 | 16.384000000000004 | 17.318 | 9.419999999999998 | 25.099999999999998 | 10.094999999999999 | 25.113000000000003 |
| 44554 | 10.25786584316492 | 18.1585 | 16.7095 | 11.847 | 25.701999999999998 | 6.926 | 27.474 |
| 44559 | 10.265333519309893 | 14.980500000000001 | 16.662999999999997 | 8.351 | 21.846000000000004 | 8.605 | 26.060999999999996 |
| 44564 | 10.286403173234486 | 15.816416666666665 | 14.507 | 9.011 | 24.029999999999998 | 5.6419999999999995 | 24.617 |
| 44569 | 10.332705177480609 | 16.404083333333336 | 17.285000000000004 | 9.26 | 25.380000000000003 | 9.782 | 26.233000000000004 |
| 44574 | 10.386690925275625 | 15.383125000000001 | 17.411 | 7.85 | 25.06 | 9.441999999999998 | 26.522000000000002 |
| 44579 | 10.453907589780865 | 14.07833333333333 | 16.9865 | 6.5 | 23.47 | 8.44 | 27.054000000000002 |
| 44584 | 10.533312026969014 | 15.301486111111108 | 16.151 | 7.459999999999999 | 25.71 | 7.023000000000001 | 26.490000000000002 |
| 44589 | 10.623749456768246 | 13.938319444444446 | 14.5535 | 6.49 | 23.86 | 3.836 | 26.619 |
| 44594 | 10.72400298155272 | 15.670347222222228 | 17.6875 | 7.85 | 25.880000000000003 | 9.806999999999999 | 26.442 |
| 44599 | 10.832838474217645 | 15.513013888888889 | 18.676499999999997 | 7.33 | 26.29 | 12.379 | 26.113999999999997 |
| 44604 | 10.949042090350249 | 18.832597222222223 | 16.3615 | 11.210000000000003 | 29.4 | 9.241 | 24.615 |
| 44609 | 11.0714488686658 | 17.394444444444442 | 18.7155 | 10.09 | 26.860000000000003 | 11.641 | 26.713 |
| 44614 | 11.198961988834826 | 18.22858333333333 | 14.717500000000001 | 11.319999999999999 | 27.389999999999997 | 8.148 | 21.551 |
| 44619 | 11.330563128503986 | 17.14626554001554 | 14.1555 | 8.56 | 27.82 | 5.220000000000001 | 24.258000000000003 |
| 44624 | 11.465314953131497 | 18.154184523809526 | 17.532 | 10.18 | 27.189999999999998 | 8.472 | 28.025 |
| 44629 | 11.602357096938334 | 17.04752777777778 | 17.7945 | 9.95 | 27.02 | 9.182 | 28.067 |
| 44634 | 11.740897102615907 | 19.232287439613533 | 19.098000000000003 | 10.75 | 28.579999999999995 | 11.783000000000001 | 27.409000000000002 |
| 44639 | 11.880197748411614 | 20.708847222222225 | 17.8645 | 11.88 | 30.079999999999995 | 9.270999999999999 | 26.878000000000004 |
| 44644 | 12.01956206910568 | 20.34040277777778 | 19.1605 | 11.790000000000001 | 30.119999999999997 | 8.85 | 30.663 |
| 44649 | 12.158317225865527 | 18.683541666666663 | 19.437 | 9.65 | 28.590000000000003 | 10.372 | 29.977999999999998 |
| 44654 | 12.295798237011022 | 18.383569444444447 | 19.353499999999997 | 10.12 | 27.639999999999997 | 9.251 | 30.165000000000003 |
| 44659 | 12.431332469397322 | 20.960277777777776 | 20.6055 | 11.78 | 31.6 | 10.257000000000001 | 31.355 |
| 44664 | 12.564225716195764 | 20.978541666666665 | 20.829 | 11.080000000000002 | 32.35 | 12.243 | 30.235000000000003 |
| 44669 | 12.693750647096895 | 21.54729166666667 | 20.315 | 11.07 | 33.040000000000006 | 10.077000000000002 | 31.148000000000003 |
| 44674 | 12.819138397373868 | 23.23034722222222 | 21.9995 | 12.069999999999999 | 35.010000000000005 | 11.942 | 32.214 |
| 44679 | 12.939574041174694 | 22.755208333333336 | 24.027000000000005 | 13.98 | 32.04 | 13.062999999999999 | 34.823 |
| 44684 | 13.05419664487863 | 22.45888888888889 | 22.020999999999997 | 12.37 | 33.029999999999994 | 12.548 | 31.459999999999997 |
| 44689 | 13.162104489145623 | 23.545138888888886 | 23.83 | 14.079999999999998 | 33.199999999999996 | 13.507 | 34.168 |
| 44694 | 13.26236585635412 | 23.91493055555556 | 23.158499999999997 | 13.280000000000001 | 33.55 | 14.228 | 32.410000000000004 |Supplementary Figure S1. Day length and average, min., and max. daily temperature during the crop cycles 2015-16, 2016-17 for HiBAP I and 2017-18 and 2018-19 for HiBAP II. DTInB: Days to initiation of booting, DTA: Days to Anthesis, DTM: Days to maturity, VGP: Vegetative growth period, GFD: Grain filling duration.
