# Supplementary material for: Effect of Flowering Time-Related Genes on Biomass, Harvest Index, and Grain Yield in CIMMYT Elite Spring Bread Wheat
Source: Biology (Basel). 2021 Sep 1;10(9):855. doi: 10.3390/biology10090855 (PMC8471161; doi:10.3390/biology10090855)
Supplement: Supplementary file 1 [file biology-10-00855-s001.zip › Suppl. References.pdf]

1. Hu, P.; Chapman, S.C.; Dreisigacker, S.; Sukumaran, S.; Reynolds, M.; Zheng, B. Using a Gene-Based Phenology Model to Identify Optimal Flowering Periods of Spring Wheat in Irrigated Mega-Environments. *J. Exp. Bot.* **2021**, doi:10.1093/jxb/erab326.
2. Chen, Y.; Carver, B.F.; Wang, S.; Cao, S.; Yan, L. Genetic Regulation of Developmental Phases in Winter Wheat. *Mol. Breed.* **2010**, *26*, 573–582, doi:10.1007/s11032-010-9392-6.
3. Milec, Z.; Tomková, L.; Sumíková, T.; Pánková, K. A New Multiplex PCR Test for the Determination of Vrn-B1 Alleles in Bread Wheat (Triticum Aestivum L.). *Mol. Breed.* **2011**, *30*, 317–323, doi:10.1007/s11032-011-9621-7.
4. Nitcher, R.; Pearce, S.; Tranquilli, G.; Zhang, X.; Dubcovsky, J. Effect of the Hope FT-B1 Allele on Wheat Heading Time and Yield Components. *J. Hered.* **2014**, *105*, 666–675, doi:10.1093/jhered/esu042.
5. Yan, L.; Fu, D.; Li, C.; Blechl, A.; Tranquilli, G.; Bonafede, M.; Sanchez, A.; Valárik, M.; Yasuda, S.; Dubcovsky, J. The Wheat and Barley Vernalization Gene VRN3 is an Orthologue of FT. *Proc. Natl. Acad. Sci. USA* **2006**, *103*, 19581–19586, doi:10.1073/pnas.0607142103.
